# S10-2: Promoting active mobility as a win-win opportunity for sustainable physical activity promotion

**DOI:** 10.1093/eurpub/ckae114.242

**Published:** 2024-09-26

**Authors:** Sonja Kahlmeier

**Affiliations:** Swiss Distance University of Applied Science FFHS, Switzerland

## Abstract

**Purpose:**

Promoting active mobility (e.g. cycling, walking) provides win-win opportunities but requires consideration of these often overlooked modes in transport policy and practice. This presentation will introduce the well-tested, evidence-based practical Health Economic Assessment Tool (HEAT) for Walking and Cycling.

**Project or policy description:**

The HEAT calculates: if x people walk or cycle a distance of y on most days, what is the economic value of impacts on premature mortality, taking into account effects of physical activity, air pollution and road fatalities, as well as on carbon emissions. It is developed through an open-ended, evidence- and multidisciplinary-based approach, led by the WHO and an interdisciplinary coordinating team. Each development step is done through consensus meetings with ad-hoc invited international experts from various fields. Since 2021, a globally applicable version of the HEAT is available.

HEAT has been named the “most-used tool for health-impact assessment in transport”, with scientific, policy and practice-related applications across the world. Selected case studies will be presented to illustrate different use-cases as well as its user-interface and workflow.

**Conclusions:**

Since its launch in 2009, the HEAT provides a win-win opportunity for sustainable physical activity promotion, particularly by allowing to also assess carbon-related emission reductions of different transport options on a population level.

The HEAT fosters advocating for transport-solutions that prioritize active transport by using an economic approach customarily used in transport planning.

**Funding:**

The HEAT has been supported by a range of donors, the WHO Regional Office for Europe and WHO Headquarters as well as European Union research projects. A full list of donors can be found on www.heatwalkingcycling.org.

